# Para-Toluenesulfonamide Induces Anti-tumor Activity Through Akt-Dependent and -Independent mTOR/p70S6K Pathway: Roles of Lipid Raft and Cholesterol Contents

**DOI:** 10.3389/fphar.2018.01223

**Published:** 2018-11-13

**Authors:** Jui-Ling Hsu, Wohn-Jenn Leu, Lih-Ching Hsu, Shih-Ping Liu, Nan-Shan Zhong, Jih-Hwa Guh

**Affiliations:** ^1^School of Pharmacy, College of Medicine, National Taiwan University, Taipei, Taiwan; ^2^Department of Urology, College of Medicine, National Taiwan University Hospital, Taipei, Taiwan; ^3^State Key Laboratory of Respiratory Disease, National Clinical Research Center for Respiratory Disease, Guangzhou Institute for Respiratory Health, First Affiliated Hospital of Guangzhou Medical University, Guangzhou, China

**Keywords:** para-toluenesulfonamide, Akt/mTOR/p70S6K pathway, lipid raft, cholesterol, castration-resistant prostate cancer

## Abstract

Castration-resistant prostate cancer (CRPC) cells can resist many cellular stresses to ensure survival. There is an unmet medical need to fight against the multiple adaptive mechanisms in cells to achieve optimal treatment in patients. Para-toluenesulfonamide (PTS) is a small molecule that inhibited cell proliferation of PC-3 and DU-145, two CRPC cell lines, through p21- and p27-independent G1 arrest of cell cycle in which cyclin D1 was down-regulated and Rb phosphorylation was inhibited. PTS also induced a significant loss of mitochondrial membrane potential that was attributed to up-regulation of both Bak and PUMA, two pro-apoptotic Bcl-2 family members, leading to apoptosis. PTS inhibited the phosphorylation of m-TOR, 4E-BP1, and p70S6K in both cell lines. Overexpression of constitutively active Akt rescued the inhibition of mTOR/p70S6K signaling in PC-3 cells indicating an Akt-dependent pathway. In contrast, Akt-independent effect was observed in DU-145 cells. Lipid rafts serve as functional platforms for multiple cellular signaling and trafficking processes. Both cell lines expressed raft-associated Akt, mTOR, and p70S6K. PTS induced decreases of expressions in both raft-associated total and phosphorylated forms of these kinases. PTS-induced inhibitory effects were rescued by supplement of cholesterol, an essential constituent in lipid raft, indicating a key role of cholesterol contents. Moreover, the tumor xenograft model showed that PTS inhibited tumor growth with a T/C (treatment/control) of 0.44 and a 56% inhibition of growth rate indicating the *in vivo* efficacy. In conclusion, the data suggest that PTS is an effective anti-tumor agent with *in vitro* and *in vivo* efficacies through inhibition of both Akt-dependent and -independent mTOR/p70S6K pathways. Moreover, disturbance of lipid raft and cholesterol contents may at least partly explain PTS-mediated anti-tumor mechanism.

## Introduction

Prostate cancer has been recognized as one of the most important medical difficulties in men. It eventually develops to castration-resistant prostate cancer (CRPC) when advanced prostate cancer progresses and metastasis appears in spite of medical treatment with androgen deprivation therapy. New therapeutic agents are needed in CRPC treatment since the patients currently have few treatment options. Given that prostate cancer cells can adapt to many cellular stresses, promising therapies targeting prostate cancer differently to fight against multiple adaptive mechanisms are highly required to offer alternative therapeutic options (Mohammad et al., 2015; Wolf, 2017). Androgen-deprivation therapy is the mainstay therapy for advanced metastatic prostate cancer; however, it ultimately progresses to CRPC. Several mechanisms have been identified to be responsible for CRPC occurrence including amplification or point mutations in androgen receptor gene, interaction between androgen receptors and growth factors, and activation of compensatory survival signaling pathways (Chen et al., 2004; Velcheti et al., 2008). The phosphoinositide 3-kinase (PI3K)/Akt signaling pathway that plays a key role in regulating cell survival and neoplastic transformation is constitutively activated in most of the CRPCs. Activation of PI3K/Akt is the most frequently reported in the category of compensatory survival signaling pathways in CRPC (Cohen-Solal et al., 2015; McCubrey et al., 2015). Tumor suppressor PTEN (phosphatase and tensin homolog deleted on chromosome 10), which is a negative regulator of PI3K/Akt activity, is mutated or lost in 50–80% patients with prostate adenocarcinoma (Velcheti et al., 2008). The decreased PTEN capability and increased PI3K/Akt activity are well correlated to a high Gleason score and with advanced pathological stage disease (McMenamin et al., 1999). Furthermore, loss of PTEN expression was correlated with worse survival and shorter time on prostate cancer therapy, such as abiraterone treatment (Ferraldeschi et al., 2015).

Furthermore, Akt can negatively regulate forkhead box transcription factor FOXO3A (a tumor suppressor) through posttranslational modifications, leading to increased cytoplasmic accumulation while decreased DNA binding which ultimately induces cell survival. It has been noted in prostate tumor specimens in which profound cytoplasmic accumulation of FOXO3A is detected with increasing Gleason score (Shukla et al., 2009). Altogether, these studies suggest a key role of PI3K/Akt in CRPC progression.

Cholesterol is an essential structural constituent to maintain the integrity and fluidity in cell membranes and is critical to synthesis of hormones, vitamin D and bile acid, and to regulation of multiple cellular signaling (Incardona and Eaton, 2000). Lipid rafts, which are membrane microdomains, preferentially associate with cholesterol, saturated lipids and kinases in regulating a number of cellular signaling pathways (Gajate and Mollinedo, 2015; Sezgin et al., 2017). Numerous studies have demonstrated that reduction or depletion of cholesterol from plasma membranes is capable of disrupting PI3K/Akt signal transduction (Huang, 2014; Yamaguchi et al., 2015), suggesting the importance of membrane cholesterol content and lipid raft integrity. Notably, breast and prostate cancer cells have been reported to be more abundant in lipid rafts which lead to their higher susceptibility to apoptotic stimuli caused by cholesterol depletion (Li et al., 2006).

Para-toluenesulfonamide (PTS) is a small molecule against several cancers including hepatocellular carcinoma, non-small cell lung cancer and tongue squamous cell carcinoma in both *in vitro* and *in vivo* studies (He et al., 2012; Gao et al., 2013; Liu et al., 2015). Furthermore, it displays efficient anti-tumor activity against advanced hepatocellular carcinoma and non-small cell lung cancer in clinical trials through a concurrent local injection therapy (He et al., 2009, 2012). PTS can penetrate to and distribute over tumors more easily because of good lipophilicity. Recent studies have demonstrated that PTS induces lysosomal membrane permeabilization and lysosomal damage, leading to cathepsin B release and activation of lysosome-mediated cell death (Liu et al., 2015). In this study, we have documented the crucial roles of lipid rafts and cholesterol in PTS-mediated redistribution and activity of several survival kinases in CRPC cells. We show the first time that the disturbance of cholesterol contents and alterations of lipid raft-associated Akt/mTOR/p70S6K pathways are responsible for PTS-induced anti-CRPC effects.

## Materials and Methods

### Materials

Human prostate adenocarcinoma cell lines, PC-3 and DU-145, were obtained from American Type Culture Collection (Rockville, MD, United States). RPMI 1640 medium, fetal bovine serum (FBS), penicillin and streptomycin were purchased from GIBCO/BRL Life Technologies (Grand Island, NY, United States). Antibodies of PARP-1, Bcl-2, Bak, Mcl-1, p53 upregulated modulator of apoptosis (PUMA), α-tubulin, cyclin E, cyclin A, cyclin B, cyclin-dependent kinase (Cdk) 4, Cdk2, Cdk1, GAPDH, p27, caveolin-1 were obtained from Santa Cruz Biotechnology, Inc. (Santa Cruz, CA, United States). Antibodies of Rb, p-Rb^Ser807/811^, p21, Akt, p-Akt^Thr308^, p-Akt^Ser473^, Bid, cyclin D1, mTOR, p-mTOR^Ser2448^, 4E-BP1, p-4E-BP1^Thr37/46^, p-p70S6K^Thr389^, and p-IκB-α^Ser32^ were from Cell Signaling Technologies (Boston, MA, United States). P70S6K was from Abcam (Cambridge, United Kingdom). Caspase-3 was from Imgenex, Corp. (San Diego, CA, United States). Carboxyfluorescein succinimidyl ester (CFSE) was from Molecular Probes Inc. (Eugene, OR, United States). Anti-mouse and anti-rabbit IgGs were from Jackson ImmunoResearch Laboratories, Inc. (West Grove, PA, United States). Para-toluenesulfonamide (PTS), sulforhodamine B (SRB), leupeptin, NaF, NaVO_4_, dithiothreitol, phenylmethylsulfonylfluoride (PMSF), trichloroacetic acid (TCA), mitoxantrone, water-soluble cholesterol, propidium iodide (PI) and all other chemical compounds were purchased from Sigma-Aldrich (St. Louis, MO, United States).

### Cell Culture

PC-3 and DU-145 cells were cultured in RPMI 1640 medium supplemented with 5% FBS (v/v), penicillin (100 units/ml) and streptomycin (100 μg/ml). Cultures were maintained in a 37°C incubator with 5% CO_2_. Adherent cultures were passaged using 0.05% trypsin-EDTA after reaching 80% confluence.

### SRB and Clonogenic Assays

Cells were seeded in 96-well plates in culture medium with 10% FBS. After 24 h, cells were fixed with 10% TCA to represent cell population at the time of compound addition (TZ). After incubation of DMSO or the compound for 48 or 72 h, cells were fixed with 10% TCA, and SRB at 0.4% (w/v) in 1% acetic acid was added for staining. Unbound SRB was washed with 1% acetic acid and SRB bounded cells were solubilized with 10 mM Tris. Absorbance was examined at 515 nm wavelengths. Growth inhibition of 50% (IC_50_) was determined at the compound concentration resulting in 50% reduction of total protein increase in control cells. To examine anchorage-dependent clonogenic effect, cells were seeded in 6-well plates. After 10-day treatment with the compound, cell colonies were rinsed with phosphate-buffered saline (PBS), stained with 0.4% (w/v) crystal violet/20% methanol and lysed by 50 mM sodium citrate/50% ethanol. The absorbance was read at 595 nm wavelengths.

### Cell Proliferation Assay With CFSE Staining

Carboxyfluorescein succinimidyl ester was dissolved in DMSO (10 mM) and was kept at -20°C until use. The cells were adjusted to 10^6^ cells/ml and treated with CFSE (10 μM). After incubation at 37°C for 10 min, labeling was blocked by RPMI medium with 10% FBS. The mixture was placed in ice for 5 min and washed. After centrifugation, cells were seeded in RPMI medium with 10% FCS with or without the compound for 48 h at 37°C under 5% CO_2_/95% air. The fluorescence intensity was determined by flow cytometry. Cell proliferation was assessed by monitoring the decrease in label intensity in daughter cells. The proliferation index and cell populations of parent or different generations were calculated by using Modfit LT Version 3.2 and WinList Version 5.0 software.

### Flow Cytometric Assay With PI Staining

Cells were harvested by trypsinization, fixed with 70% (v/v) alcohol at 4°C for 30 min and washed with PBS. After centrifugation, cells were incubated in phosphate-citric acid buffer (pH: 7.8) for 30 min at room temperature. The cells were centrifuged and re-suspended with 0.5 ml PI solution containing Triton X-100 (0.1% v/v), RNase (100 μg/ml) and PI (80 μg/ml). DNA content was analyzed with the FACScan and CellQuest software (Becton Dickinson, Mountain View, CA, United States).

### Western Blotting

After treatment, cells were harvested with trypsinization, centrifuged and lysed in 0.1 ml of lysis buffer containing 10 mM Tris-HCl (pH 7.4), 150 mM NaCl, 1 mM EGTA, 1% Triton X-100, 1 mM PMSF, 10 μg/ml leupeptin, 10 μg/ml aprotinin, 50 mM NaF and 100 μM sodium orthovanadate. Total protein was quantified, mixed with sample buffer and boiled at 90°C for 5 min. Equal amount of protein (30 μg) was separated by electrophoresis in 8% or 12% SDS-PAGE, transferred to PVDF membranes and was detected with specific antibodies (1:1000 dilution). The immunoreactive proteins after incubation with appropriately labeled secondary antibody (1:3000 dilution) were detected with an enhanced chemiluminescence detection kit (Amersham, Buckinghamshire, United Kingdom).

### Measurement of Mitochondrial Membrane Potential (ΔΨ_m_)

JC-1, a mitochondrial dye staining mitochondria in living cells in a membrane potential-dependent fashion, was used to determine ΔΨ_m_. Cells were treated with or without the compound. Thirty minutes before termination of incubation, cells were incubated with JC-1 (5 μM) at 37°C for 10 min. Accumulation of JC-1 was determined using flow cytometry.

### Transient Transfection

The plasmid encoding Myr-Akt, an N-terminally myristoylation signal-attached Akt (courtesy of Prof. Mien-Chie Hung, The University of Texas, M. D. Anderson Cancer Center). PC-3 cells were seeded into 60-mm tissue culture dishes with 30% confluence and grown for 24 h to 50–60% confluence. Each dish was washed with serum-free Opti-MEM (Life Technologies), and 2 ml of the same medium was added. Aliquots containing Myr-Akt expression vector or a control plasmid in serum-free Opti-MEM were transfected into cells using Lipofectamine 2000 (Invitrogen). After incubation for 6 h at 37°C, cells were washed and incubated in 10% FBS-containing RPMI-1640 medium for 48 h. The cells were treated with or without the compound.

### DNA Fragmentation Assay

DNA fragmentation was determined using Cell Death Detection ELISAplus kit (Roche, Mannheim, Germany). The assay was based on quantitative *in vitro* determination of cytoplasmic histone-related DNA fragments (mono- and oligonucleosomes). After treatment with the compound, the cells were lysed and centrifuged, and the supernatant was used for detection of nucleosomal DNA.

### Lipid Raft Isolation

Lipid rafts were isolated using lysis conditions and centrifugation on discontinuous sucrose gradients. Briefly, after treatment, the cells were washed with ice-cold PBS and lysed for 30 min on ice with 1% Triton X-100 in TNEV buffer (10 mM Tris-HCl, pH 7.5, 150 mM NaCl, 5 mM EDTA, 1 mM Na_3_VO_4_, 1 mM PMSF). Cells were homogenized with Biovision tissue homogenizer. After centrifugation (200 *g*, 8 min), the nuclei and cellular debris were pelleted and the supernatant (400 μl) was mixed with 400 μl 85% (w/v) sucrose in TNEV buffer, transferred to Beckman 13 mm × 51 mm centrifugal tube. The diluted lysate was overlaid with 2.4 ml 35% (w/v) sucrose in TNEV buffer and finally 1.4 ml 5% (w/v) sucrose in TNEV buffer. The samples were centrifuged in an SW55 rotor at 200,000 *g* for 18 h at 4°C in an ultracentrifuge (Beckman Instruments, Palo Alto, CA, United States). The fractions (350 μl each) were collected from the highest gradient. 15 μl of each fraction was subjected to Western blot analysis.

### *In vivo* Anti-tumor Study

PC-3-derived cancer xenografts in nude mice were used as an *in vivo* model. The nude mice were subcutaneously injected with PC-3 cells (10^7^ cells/mouse). When the tumor volume reached 100 mm^3^, the mice were divided into two groups (*n* = 8–10) and compound treatment was initiated. PTS was dissolved in 15% 1-Methyl-2-pyrrolidone (NMP). Vehicle (15% NMP) or PTS was injected intraperitoneally every other day. The tumor length (*l*) and width (*w*) were measured, and tumor volume was calculated as *lw*^2^/2. The protocols of the *in vivo* study were approved by the Animal Care and Use Committee at National Taiwan University. All animal procedures and protocols were approved by AAALAC-accredited facility.

### Data Analysis

Data were presented as mean ± SD. Statistical analysis was performed and two-group comparisons were done with Student’s *t*-test. *P* < 0.05 was considered statistically significant.

## Results

### PTS Inhibits Cell Proliferation in CRPC Cells

**FIGURE 1:**
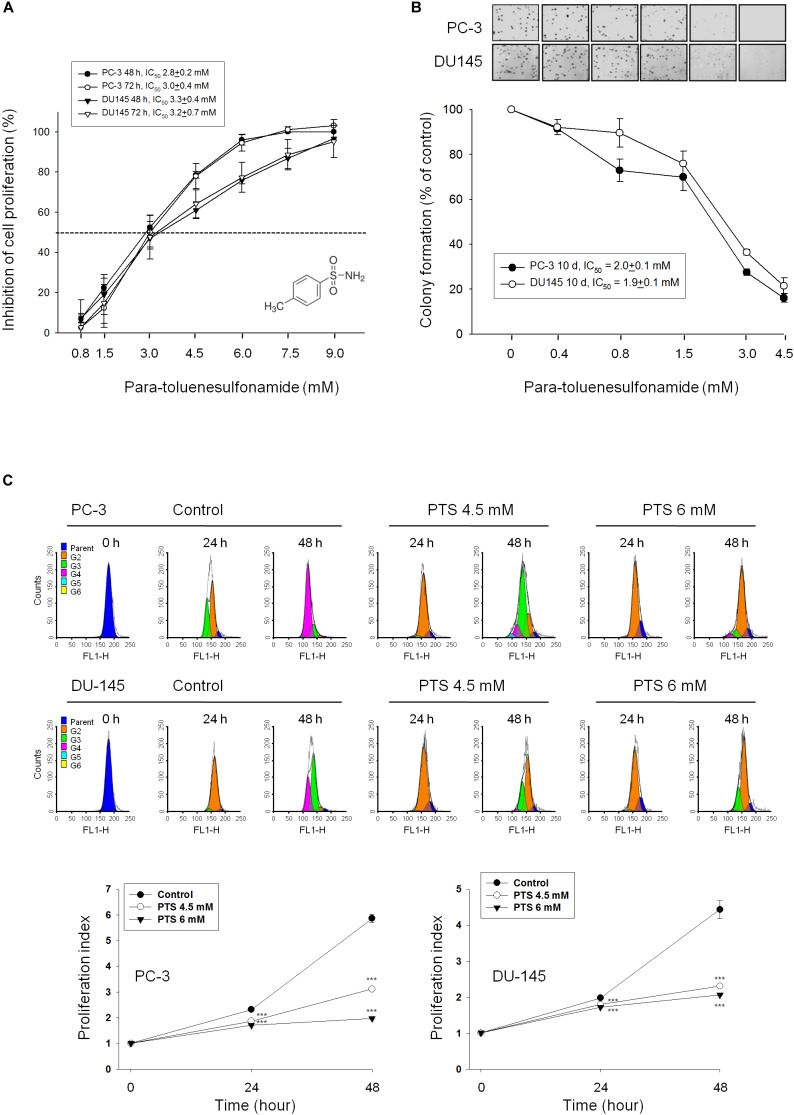
Anti-proliferative effects of para-toluenesulfonamide (PTS) on human castration-resistant prostate cancer cells. The cells were incubated in the absence or presence of PTS for different duration. After treatment, cells were fixed and stained for SRB assay **(A)** and colony formation assay **(B)**. **(C)** PC-3 and DU-145 cells were incubated with or without PTS. After treatment, cells were harvested for flow cytometric analysis of CFSE staining. The proliferation index and the cell populations of parent or different generations were calculated by Modfit LT Version 3.2 and WinList Version 5.0 software. Quantitative data are expressed as mean ± SD of three to four independent experiments. ^∗∗∗^*P* < 0.001 compared with the control.

Sulforhodamine B assay, an accurate and reproducible assay based upon quantitative SRB staining of cellular proteins, was used for anti-proliferative determination in this study. PTS showed a concentration-dependent inhibition of both PC-3 and DU-145 cell lines with IC_50_ values around 3 mM (Figure 1A). Furthermore, the data in clonogenic assay demonstrated that PTS displayed a long-term anti-proliferative effect (10 days) in both PC-3 and DU-145 cells (Figure 1B). The anti-proliferative effect was further examined by CFSE staining, a cell-tracking dye, which conjugated to intracellular proteins and was evenly inherited by divided cells after cell proliferation. Consequently, the fluorescence-staining was distributed to later generations of cells with the passage of time. PTS significantly inhibited cell proliferation, inducing an increase of cell population in earlier generations. The proliferation index in both PC-3 and DU-145 cells based on CFSE staining assay showed a concentration-dependent inhibition to PTS action (Figure 1C). Because PTS is a simple small molecule with a molecular weight of 171 da, it is reasonable that PTS is effective with concentrations in millimolar range. Several compounds and drugs also have been reported to display activities at millimolar concentrations, such as *N-acetylcysteine and trolox* in scavenging reactive oxygen species (ROS) and aspirin and epigallocatechin-3-gallate in inducing cell-cycle arrest and apoptotic cell death (Arango et al., 2001; Lee et al., 2015).

### PTS Induces G1 Phase Arrest and Mitochondrial Stress

**FIGURE 2:**
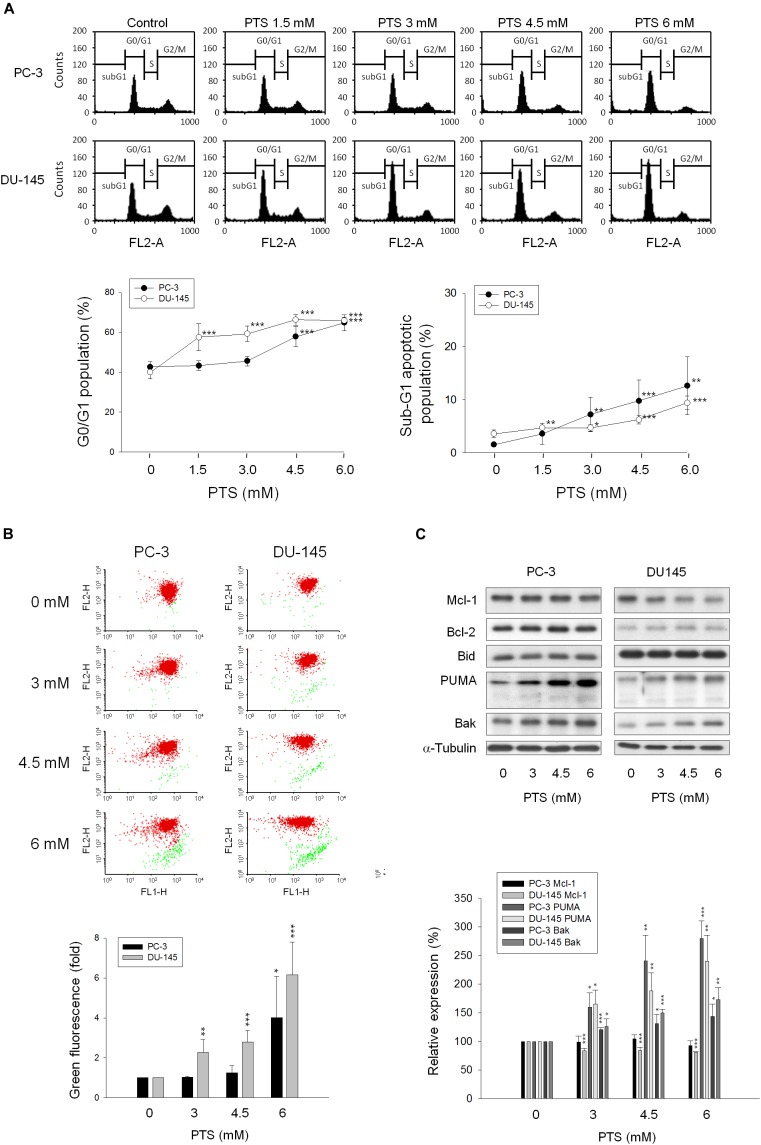
Effect of PTS on cell cycle arrest and mitochondrial dysfunction. PC-3 and DU-145 cells were incubated in the absence or presence of PTS for 24 **(A,C)** and 48 h **(B)**. The cells were harvested for propidium iodide staining to analyze the distribution of cell populations in cell cycle phases **(A)**, or for JC-1 staining to detect mitochondrial membrane potential using FACScan flow cytometric analysis **(B)**. The green fluorescence was shown for the quantification of mitochondrial membrane potential **(B)**. **(C)** The cells were harvested and lysed for the detection of protein expressions of several Bcl-2 family members by Western blot analysis. Data are expressed as mean ± SD of three to five determinations. ^∗^*P* < 0.05, ^∗∗^*P* < 0.01 and ^∗∗∗^*P* < 0.001 compared with the control.

**FIGURE 3:**
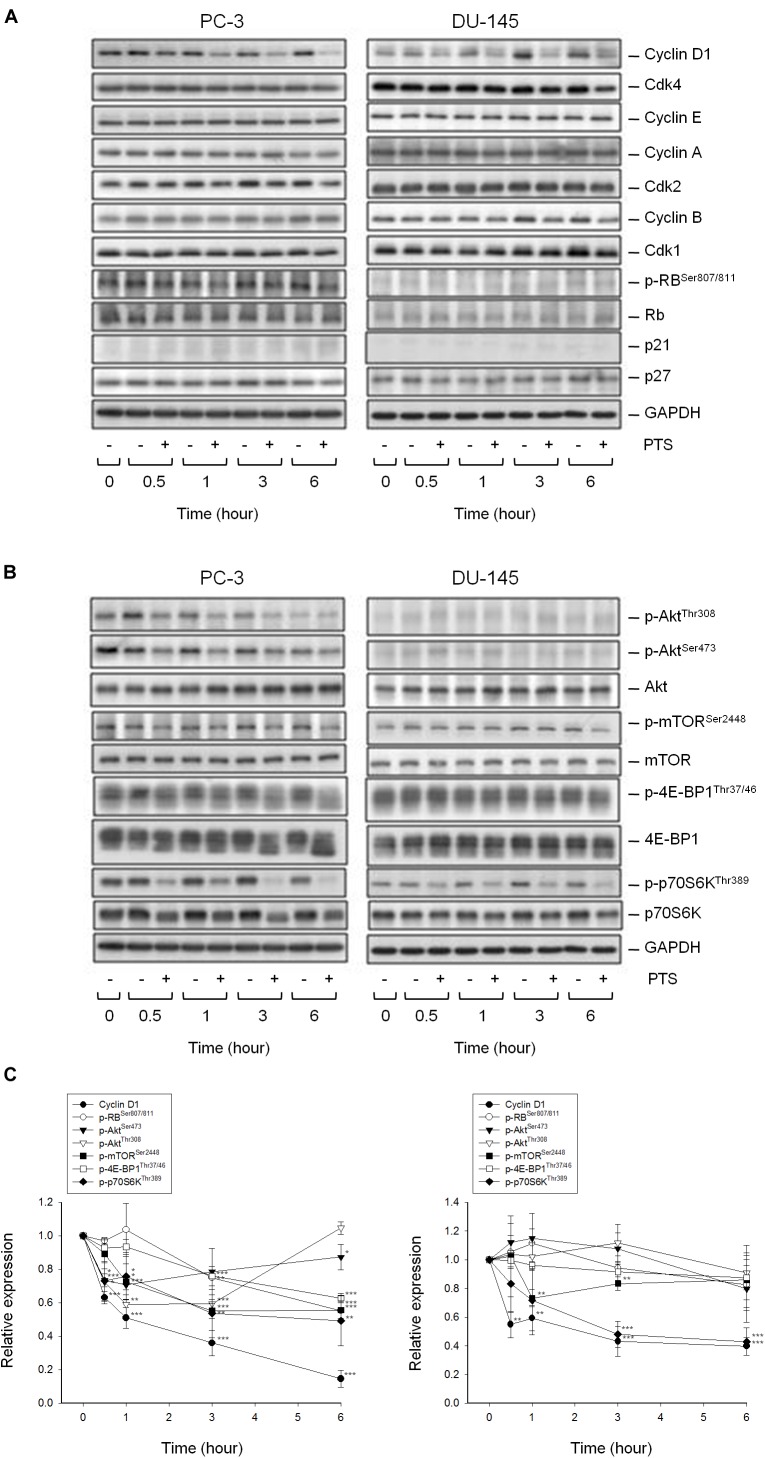
Effect of PTS on the expression of cell cycle regulators and kinases. The cells were incubated in the absence or presence of 6 mM PTS for the indicated times. After treatment, the cells were harvested and lysed for the detection of protein expressions of cell cycle regulators **(A)**, and Akt/mTOR/p70S6K pathway signals **(B)** by Western blot analysis. The control GAPDH images are re-used in Figures 3A,B because they are the same experiment. The expression was quantified using Image Lab Software 6.0 (BIO-RAD) **(C)**. Data are expressed as mean ± SD of three determinations. ^∗^*P* < 0.05, ^∗∗^*P* < 0.01, ^∗∗∗^*P* < 0.001 compared with the control.

As shown in Figure 2A, PTS induced accumulation of both PC-3 and DU-145 cells in G1 phase and accelerated cell apoptosis. When cellular stress occurs, G1 phase arrest takes place until cellular damage is fixed. If not properly repaired, apoptosis can be triggered through the inhibition of pro-survival components or the activation of apoptotic pathways in which mitochondria are the most sensitive organelles to orchestrate these signals. The data demonstrated that PTS resulted in a concentration-dependent decrease of mitochondrial membrane potential (Figure 2B), indicating that mitochondrial stress led to caspase-dependent apoptosis because Z-VAD-FMK, a pan-caspase inhibitor, profoundly inhibited PTS-induced apoptosis using both flow cytometric analysis of PI staining and nucleosomal DNA fragmentation assay (Supplementary Figure S1). Mitochondrial membrane permeability is directly controlled by Bcl-2 family of proteins (Wolf, 2017; Lee et al., 2018). PTS increased the expressions of PUMA and Bak, two pro-apoptotic Bcl-2 family members, in both PC-3 and DU-145 cells (Figure 2C). Additionally, PTS suppressed Mcl-1 expression, an anti-apoptotic Bcl-2 family member, in DU-145 cells (Figure 2C). In G1 phase, cyclin D1/CDK4 complex is responsible for progression to S phase by the phosphorylation of Rb protein. PTS decreased cyclin D1 protein expression and Rb phosphorylation in PC-3 cells, but only a decrease of cyclin D1 expression was observed in Rb-mutant DU-145 cells. Notably, neither p21 nor p27 expressions were modified by PTS in both cell lines (Figure 3A).

### PTS Inhibits Akt/mTOR/p70S6K Activation

**FIGURE 4:**
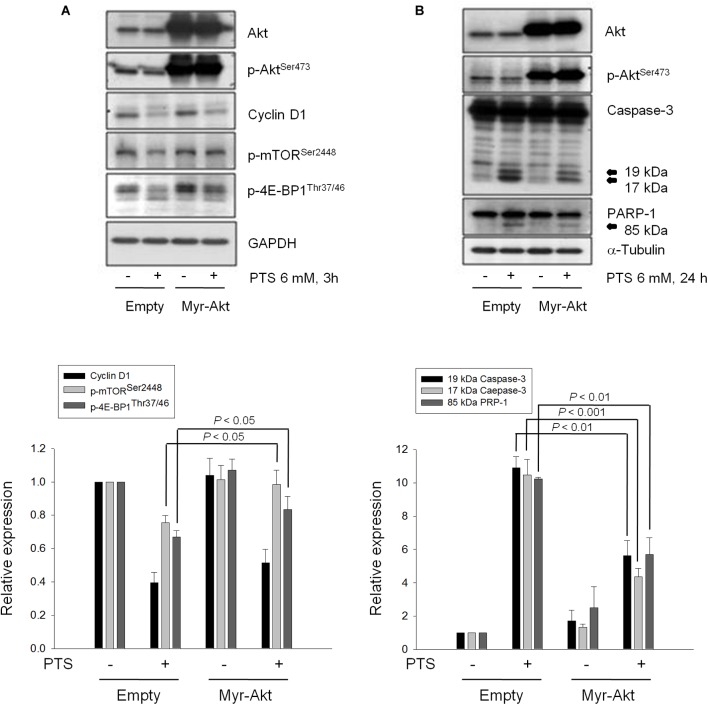
Effect of Akt on PTS-induced alteration in several protein expressions. PC-3 cells were transfected with Myr-Akt plasmid. Then, the cells were incubated in the absence or presence of PTS for 3 **(A)** or 24 h **(B)**. The cells were harvested and lysed for the detection of the indicated protein by Western blot analysis. The expression was quantified using Image Lab Software 6.0 (BIO-RAD). The data are expressed as mean ± SD of three independent experiments.

The serine/threonine kinases mTOR and p70S6K, which regulate protein synthesis through affecting the phosphorylation or activities of several downstream translation factors, are critical regulators in G1 phase (Asnaghi et al., 2004; Fenton and Gout, 2011). PTS induced an inhibitory effect on the phosphorylation of mTOR, 4E-BP1 (a repressor of mRNA translation) and p70S6K in both PC-3 and DU-145 cells. Moreover, PTS inhibited the phosphorylation of Akt, a critical player of signaling pathways of mTOR, 4E-BP1 and p70S6K in PC-3 cells. In contrast, Akt phosphorylation was not modified by PTS in DU-145 cells, suggesting an Akt-independent mTOR/p70S6K signaling (Figures 3B,C). To further substantiate the role of Akt, we have identified that overexpression of constitutively active Myr-Akt in PC-3 cells significantly rescued the inhibitory effects on both mTOR and 4E-BP1 phosphorylation, and suppressed the activation of caspase-3 and cleavage of downstream substrate PARP-1 (Figures 4A,B). Furthermore, the functional rescue of Myr-Akt was determined. The data showed that overexpression of constitutively active Myr-Akt in PC-3 cells moderately but significantly blunted PTS-mediated growth inhibition using both SRB assay and colony formation assay (Supplementary Figure S2); the IC_50_ values were significantly shifted from 1.97 ± 0.01 to 2.18 ± 0.10 mM (*P* < 0.05) and 0.58 ± 0.02 to 0.68 ± 0.02 mM (*P* < 0.01), respectively. However, Myr-Akt overexpression had minimal effects on PTS-induced cyclin D1 down-regulation (Figure 4A).

### Lipid Raft and Cholesterol Are Crucial to the Activities of Several Kinases to PTS Action

**FIGURE 5:**
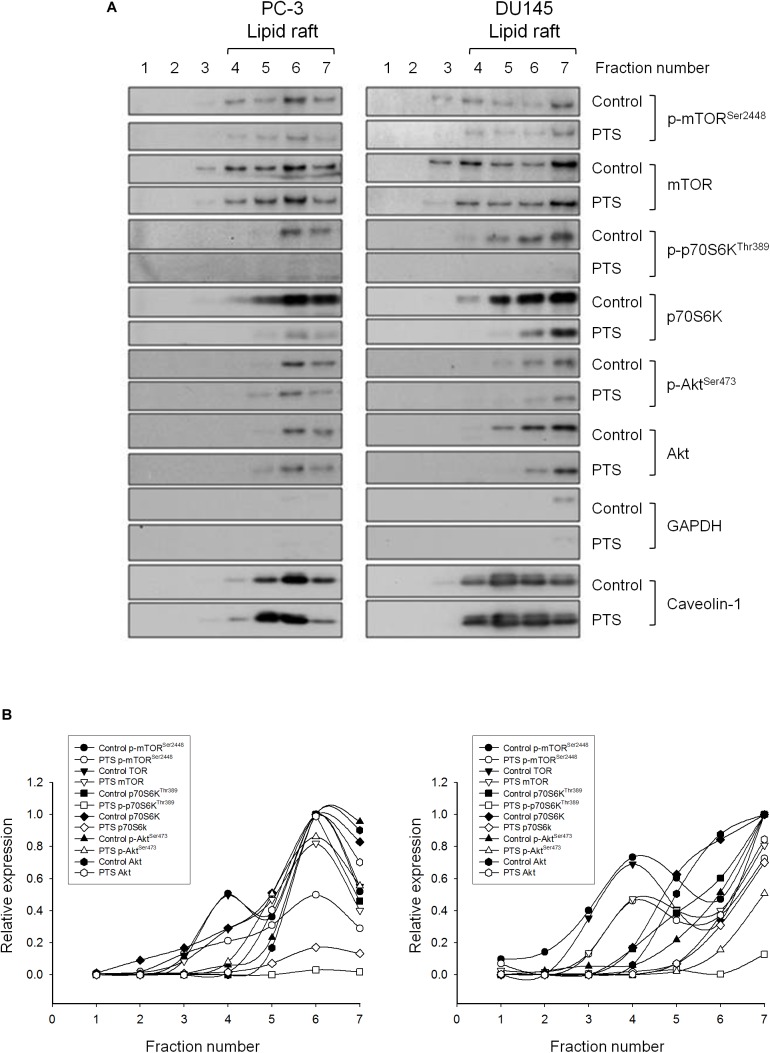
Effect of PTS on lipid raft-associated expressions of several kinases. **(A)** PC-3 and DU-145 cells were incubated in the absence or presence of 6 mM PTS for 2 h. After treatment, the cells were lysed in 1% Triton X-100 and fractionated by centrifugation as described in Section “Materials and Methods.” The protein expressions were detected by Western blotting. **(B)** The expressions were quantified using Image Lab Software 6.0 (BIO-RAD).

Lipid rafts are plasma membranes of cells containing glycosphingolipids and a number of receptors arranged in specific glycolipoprotein microdomains which serve as organizing centers to gather signaling molecules, membrane fluid and protein trafficking for a variety of cellular processes (Sezgin et al., 2017). It has been suggested that several raft-associated signaling pathway components, including Akt, mTOR and p70S6K, involve in the regulation of cell survival (Liu et al., 2014). In the present study, lipid rafts were isolated from whole cells by sucrose gradient fractionation with subsequent analysis of several serine/threonine kinases localization in raft fractions by immunoblotting. It showed that both PC-3 and DU-145 cell lines expressed all three raft-associated kinases with varied levels in which p70S6Ks were the most abundant (Figures 5A,B). PTS led to decreased expressions of both raft-associated total and phosphorylated kinases in which the phosphorylated p70S6K expressions were completely abolished in both cell lines (Figures 5A,B).

**FIGURE 6:**
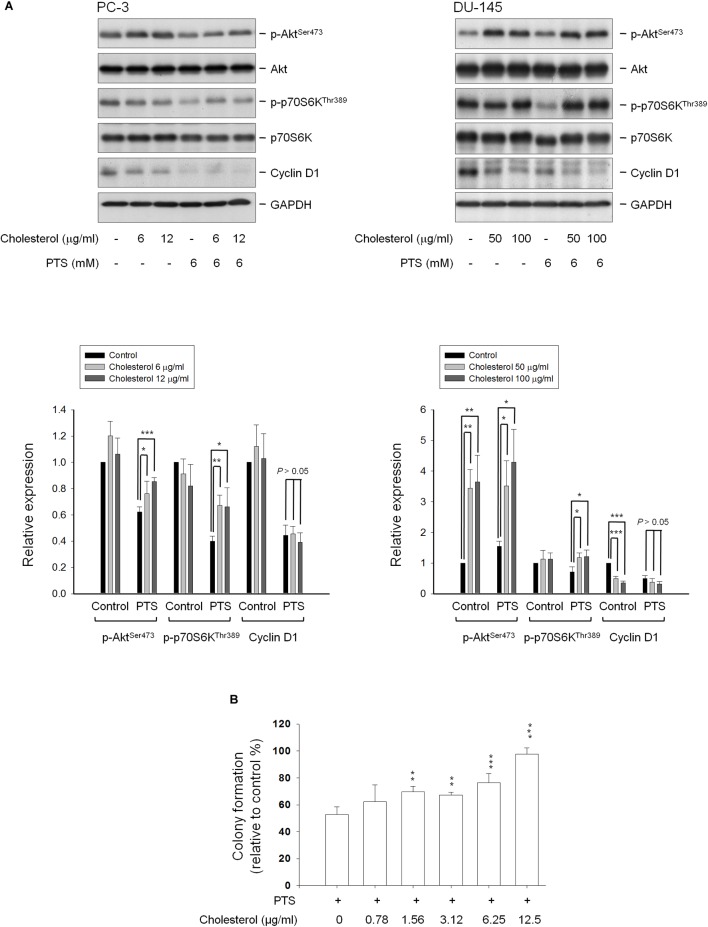
Effect of cholesterol supplement on PTS-mediated effects. **(A)** PC-3 and DU-145 cells were incubated in the absence or presence of the indicated agent for 1 h. After treatment, the cells were harvested and lysed for the detection of protein expressions by Western blot analysis. The expression was quantified using Image Lab Software 6.0 (BIO-RAD). **(B)** PC-3 cells were incubated in the presence of the indicated agent (PTS, 1.5 mM) for 10 days. After treatment, cells were fixed and stained for colony formation assay. Data are expressed as mean ± SD of three determinations. ^∗^*P* < 0.05, ^∗∗^*P* < 0.01, ^∗∗∗^*P* < 0.001 compared with PTS alone.

Cholesterol, a unique lipid molecule biosynthesized by all animal cells, is an essential structural constituent in cell membranes to maintain their structural integrity and fluidity. Rafts are composed of sphingolipids and cholesterol in outer exoplasmic leaflet, connected to phospholipids and cholesterol in inner cytoplasmic leaflet of the lipid bilayer (Simons and Ehehalt, 2002; Hsu and Guh, 2017). Recent studies have revealed that cholesterol depletion from lipid rafts is involved in apoptosis of several cancers, suggesting the modification of cellular cholesterol levels may be a potential anticancer strategy (Onodera et al., 2013; Hsu and Guh, 2017). The cholesterol function has been examined showing that proper concentrations of cholesterol supplement significantly rescued PTS-induced decrease of both Akt and p70S6K phosphorylation, but not cyclin D1 (Figure 6A) in PC-3 and DU-145 cells. Notably, cholesterol, by itself, induced an increase of Akt phosphorylation while a decrease of cyclin D1 protein expression particularly in DU-145 cells (Figure 6A). The functional rescue of cholesterol in cell growth also was determined. The data showed that cholesterol significantly rescued PTS-induced inhibition of cell growth using colony formation assay (Figure 6B).

### PTS Displays *in vivo* Efficacy in Mouse Xenograft Models

**FIGURE 7:**
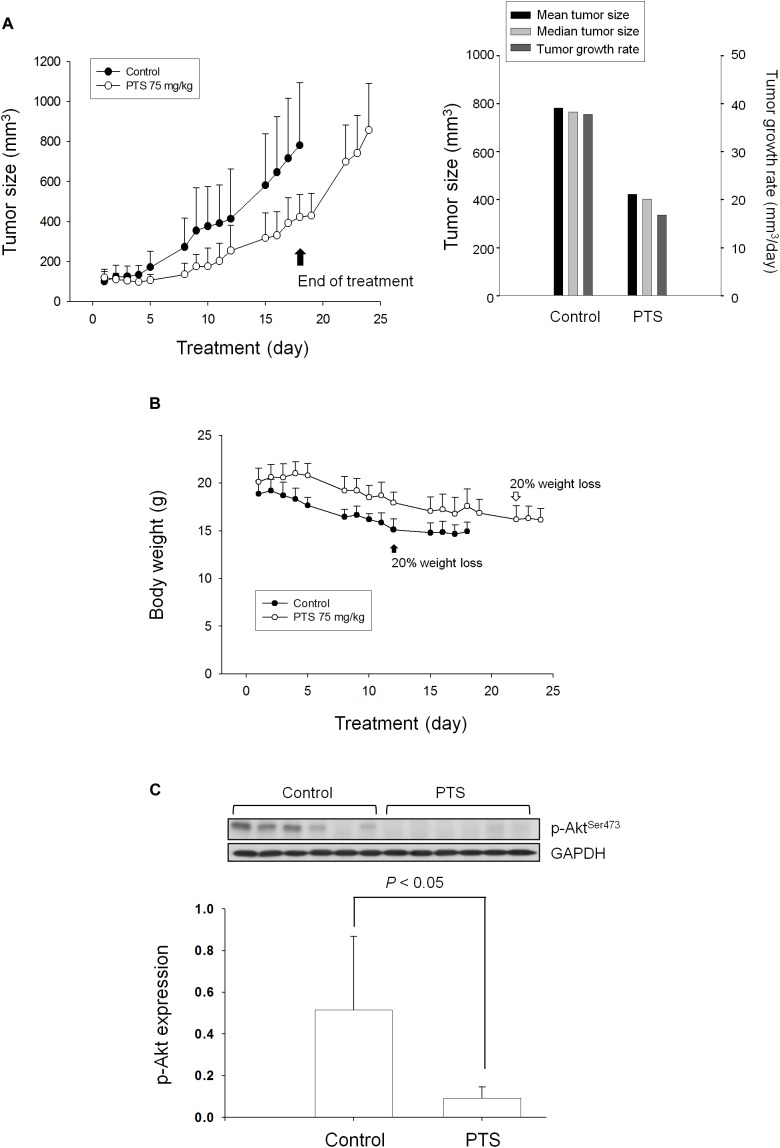
Effect of PTS in an *in vivo* anti-tumor xenograft model. The nude mice were subcutaneously injected with PC-3 cells (10^7^ cell/mouse). The tumors were measured every day. When the tumors reached to a volume of 100 mm^3^, the mice were divided into two groups and intraperitoneal PTS injection was initiated. **(A)** The length (*l*) and width (*w*) of the tumor were measured, and tumor volume was calculated as *lw*^2^/2. **(B)** The body weight was also measured. The protocols of the *in vivo* study were approved by the Animal Care and Use Committee at National Taiwan University. All animal procedures and protocols were approved by AAALAC-accredited facility. **(C)** The p-Akt expression of randomly selected six tumors in both control and PTS groups has been detected. Data are expressed as mean ± SD.

The tumor xenografts in nude mice after subcutaneously inoculated PC-3 cells were used to conduct *in vivo* efficacy evaluation. Notably, the operator was fully blinded to the experimental treatment. PTS was intraperitoneally injected when the tumor size reached to 100 mm^3^ (PTS group 121 ± 40 mm^3^ vs. control group 101 ± 48 mm^3^). PTS inhibited tumor growth with a treatment/control (T/C) ratio of 0.44 at end-of-treatment. The growth rates of control group vs. PTS group were 37.8 vs. 16.8 mm^3^/day, indicating a 56% inhibition by PTS. Furthermore, the median tumor size of PTS group was 403 mm^3^ compared with 765 mm^3^ in control group, revealing a 47% inhibition by PTS. Moreover, cessation of PTS treatment caused a rebounded growth of tumor (Figure 7A). There was a progressive loss of weight in both control and PTS groups, although no significant between-group difference was shown. The control group reached to a 20% loss of weight on the 12th day compared with the 22th day in PTS group (Figure 7B). Furthermore, the detection of p-Akt expression in tumors also showed a significant inhibitory effect of PTS (Figure 7C).

## Discussion

Autonomous cell proliferation is driven by activated survival- and growth-promoting oncogenes. PI3K/Akt/mTOR/p70S6K signaling pathway is a commonly activated pathway in prostate cancer cells. Loss of PTEN tumor suppressor is frequently reported in aberrant activation of this pathway implicated not only in survival and growth of prostate cancer cells but also in tumor metastasis (Cohen-Solal et al., 2015; Ciccarese et al., 2017; Statz et al., 2017). Inactivation of this pathway may offer opportunities for treatment of prostate cancer. Some studies have reported promising preclinical results of PI3K inhibitors while the data from clinical trials were less convincing. Accordingly, dual PI3K/mTOR inhibitors that block both PI3K/Akt and mTOR have been proposed to achieve better anti-tumor outcomes (Tang and Ling, 2014; Statz et al., 2017). However, prostate cancer which shows a wide variety of biological and clinical behavior represents the epiphenomenon of an extreme genetic heterogeneity (Ciccarese et al., 2017). The examination of multiple specific molecular alterations and the development of the most appropriate therapy based on the multiple factors may provide better opportunities for therapy.

Recently, PTS has been developed for cancer therapy (He et al., 2009, 2012; Gao et al., 2013; Liu et al., 2015). However, the anti-tumor mechanisms have not been clearly identified. We have targeted CRPC by using both PC-3 and DU-145 cell lines to elaborate PTS-mediated multiple mechanisms that efficiently block tumor growth and survival of cells both *in vitro* and *in vivo*. PTS displayed an effective and long-term stable anti-proliferative activity through induction of G1 phase that ultimately induced cell apoptosis. Typically, cellular stresses tend to cause G1 checkpoint arrest to allow cellular repair to rescue cells from programmed cell death. However, PTS induced mitochondrial damage, indicating that the cellular impairment was not significantly repaired during PTS treatment that ultimately led to apoptosis. The integrity and permeability of mitochondrial membrane are critically regulated by Bcl-2 family of proteins. PUMA is a p53-dependent and p53-independent pro-apoptotic member of BH3-only subgroup and has been identified to directly bind anti-apoptotic Bcl-2 members through its BH3 domain which induces the activation of pro-apoptotic Bcl-2 members and an increase of outer mitochondrial membrane permeability, leading to mitochondrial dysfunction and caspase activation (Hikisz and Kiliańska, 2012). Recently, several lines of evidence suggest that PUMA, similar to Bim, Noxa, and tBid, are direct Bak activators to initiate oligomerization and activation of Bak (Dai et al., 2014). PTS significantly induced an increase in expressions of PUMA and Bak, suggesting their contribution to mitochondrial dysfunction. Notably, PTS also significantly inhibited Mcl-1 expression in DU-145 cells. It has been reported that Mcl-1 and PUMA co-localize at the mitochondria and Mcl-1 level can be increased during co-expression with PUMA, indicating that PUMA can stabilize Mcl-1. In contrast, several studies have revealed that binding of PUMA to Mcl-1 is not sufficient to prevent rapid degradation of Mcl-1 (Mei et al., 2005). Consistently, we demonstrated that PUMA did not prevent Mcl-1 degradation and, furthermore, mitochondrial dysfunction was partially attributed to Mcl-1 degradation in DU-145 cells.

mTOR activity is crucial for mRNA and DNA synthesis during G1 phase. PTS markedly inhibited mTOR phosphorylation in Ser2448 within a C-terminal regulatory region, a crucial marker of mTOR activation (Sekulić et al., 2000; Asnaghi et al., 2004). Of note, it can be activated through Akt-dependent or -independent pathway (Asnaghi et al., 2004; Chiang and Abraham, 2005). The overexpression of Myr-Akt almost completely abolished PTS-mediated inhibition of phosphorylation at both mTOR^Ser2448^ and 4E-BP1^Thr37/46^ (a direct substrate of mTOR) in PC-3 cells, suggesting the inhibition of Akt-dependent mTOR activity to PTS action. In contrast, the fact that Akt activity in DU-145 cells was not apparent might be due to the presence of PTEN, a negative regulator of PI3K/Akt activity (Bastola et al., 2002).

Cyclin D1 is a key regulator in G1 phase. Aberrant cyclin D1 expression is implicated in tumorigenesis, metastasis and tumor progression in many human neoplasms (Drobnjak et al., 2000; Fusté et al., 2016). Cyclin D1 overexpression has been implicated in prostate carcinogenesis and aggravated bone metastasis (Drobnjak et al., 2000). PTS induced G1 arrest and efficiently blocked cyclin D1 expression in both bone metastasis-derived PC-3 and brain metastasis-derived DU-145 cells, indicating the potential of PTS on inhibiting metastasis in prostate cancers. However, Myr-Akt overexpression did not rescue the cyclin D1 down-regulation, indicating the existence of Akt-independent regulatory pathways. Several pathways have been proposed to be involved in cyclin D1 down-regulation, including the reduction of cellular ATP levels, activation of protein kinase C and phosphatase PP2A, depletion of adenine nucleotide translocase 2 and down-regulation of c-Myc (Guan et al., 2007; Amendola et al., 2009; Watanabe et al., 2017). The clear pathway needs further elucidation. Of note, PTS-induced G1 arrest was independent of p21 and p27. Similar effects have been previously reported. Vaziri et al. (1998) reported that butyrate-induced G1 arrest occurred in primary cultures of fibroblasts from transgenic p21 “knockout” mice (p21^-/-^) indicating the independency of p21 induction. Berns et al. (2000) reported that a dominant negative mutant of c-Myc could induce G1 arrest in embryo fibroblasts deficient for both p27 and p21. These studies support that both p21 and p27 are not rate-limiting cell cycle regulators to PTS-mediated G1 arrest.

Recently, much attention has been paid to the function of lipid raft in anticancer research since raft-associated signaling pathway components, including Akt, mTOR and p70S6K, which have been implicated in cell survival regulation (Liu et al., 2014). It is of importance in dealing with prostate cancer because prostate cancer cells contain more lipid rafts (Li et al., 2006). Cholesterol is essential in keeping membrane integrity and fluidity and is crucial for raft/caveolae formation. The changes of cholesterol contents of cells can modify the properties of lipid rafts (Simons and Gerl, 2010) and cholesterol depletion from the plasma membrane induces apoptosis, in particular in prostate cancer cells that have higher membrane cholesterol contents (Li et al., 2006). Therefore, the therapy with target for rafts/cholesterol would be a potential strategy. Our study demonstrated that both PC-3 and DU-145 cell lines expressed raft-associated Akt, mTOR, and p70S6K. PTS decreased these protein expressions in both total and phosphorylated forms. Several studies suggest that the integrity of lipid rafts is necessary to the activities of these kinases. Disturbance or disruption of lipid raft is able to impair the phosphorylation of these kinases (Calay et al., 2010; Reis-Sobreiro et al., 2013). Therefore, PTS might cause the disturbance or disruption of lipid raft, leading to the dissociation and inactivation of these kinases. The supplement of cholesterol significantly rescued PTS-induced inactivation of these kinases and further linked the role of cholesterol on PTS-mediated raft disruption. However, cholesterol supplement did not prevent the down-regulation of cyclin D1 because it was not raft-associated component. The data also supported the notion that cyclin D1 down-regulation was not downstream event of Akt/mTOR/p70S6K pathway. Finally, nude mice xenograft model was used to examine the *in vivo* anti-tumor efficacy. Intraperitoneal treatment of PTS induced a 56% inhibition of tumor growth through the measurement of T/C and growth rate and a 47% inhibition by detecting median tumor size. Akt phosphorylation in tumors also was significantly inhibited by PTS. The data revealed an *in vivo* efficacy of PTS.

It is noteworthy that the use of statins, HMG-CoA reductase inhibitors on lowering lipid, has been reported to be clinically associated with prolonged overall survival and cancer-specific survival in patients with metastatic CRPC receiving abiraterone or enzalutamide although future validation is warranted (Gordon et al., 2018). The research supports the potential of the combination treatment of CRPC patients with PTS and therapeutic drugs. Furthermore, the PTS activity may be enhanced by statins based on the distinct mechanisms on lowering lipid. However, prospective elucidation is warranted.

## Conclusion

**FIGURE 8:**
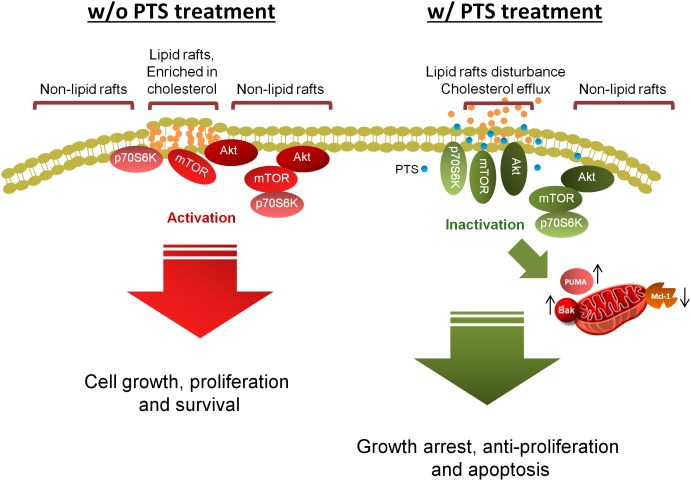
Schematic figure for PTS-mediated signaling pathways. PTS induces anticancer effect through an arrest of the cell cycle at G1 phase and apoptosis *via* down-regulation of Mcl-1 and up-regulation of both Bak- and PUMA which induce mitochondrial dysfunction in CRPC cells. Furthermore, both Akt-dependent and -independent mTOR/p70S6K pathway are involved in PTS-mediated pathways. Disturbance of lipid raft and cholesterol contents may, at least partly, explain the dissociation and inactivation of Akt, mTOR, and p70S6K in CRPC cells.

The data suggest that PTS is an effective anti-tumor agent with both *in vitro* and *in vivo* efficacies (Figure 8). PTS induces anti-proliferative effect through an arrest of the cell cycle at G1 phase and apoptosis *via* Bak- and PUMA-involved mitochondrial dysfunction in both PC-3 and DU-145 cells. Furthermore, Akt is critical to mTOR/p70S6K pathway in the apoptotic regulation in PTEN null/Akt active PC-3 cells but not in PTEN wild-type DU-145 cells. Notably, disturbance of lipid raft and cholesterol contents may, at least partly, explain the dissociation and inactivation of Akt, mTOR and p70S6K in both cell lines.

## Author Contributions

N-SZ and J-HG contributed to the conception and design of the experiments. J-LH performed the experiments and analyzed the data. W-JL, L-CH, and S-PL participated the progress reports and troubleshooting in experiments. N-SZ and J-HG wrote the manuscript.

## Conflict of Interest Statement

The authors declare that the research was conducted in the absence of any commercial or financial relationships that could be construed as a potential conflict of interest.
