# Supplementary figures and images for: Para-Toluenesulfonamide Induces Anti-tumor Activity Through Akt-Dependent and -Independent mTOR/p70S6K Pathway: Roles of Lipid Raft and Cholesterol Contents
Source: Front Pharmacol. 2018 Nov 13;9:1223. doi: 10.3389/fphar.2018.01223 (PMC6282052; doi:10.3389/fphar.2018.01223)

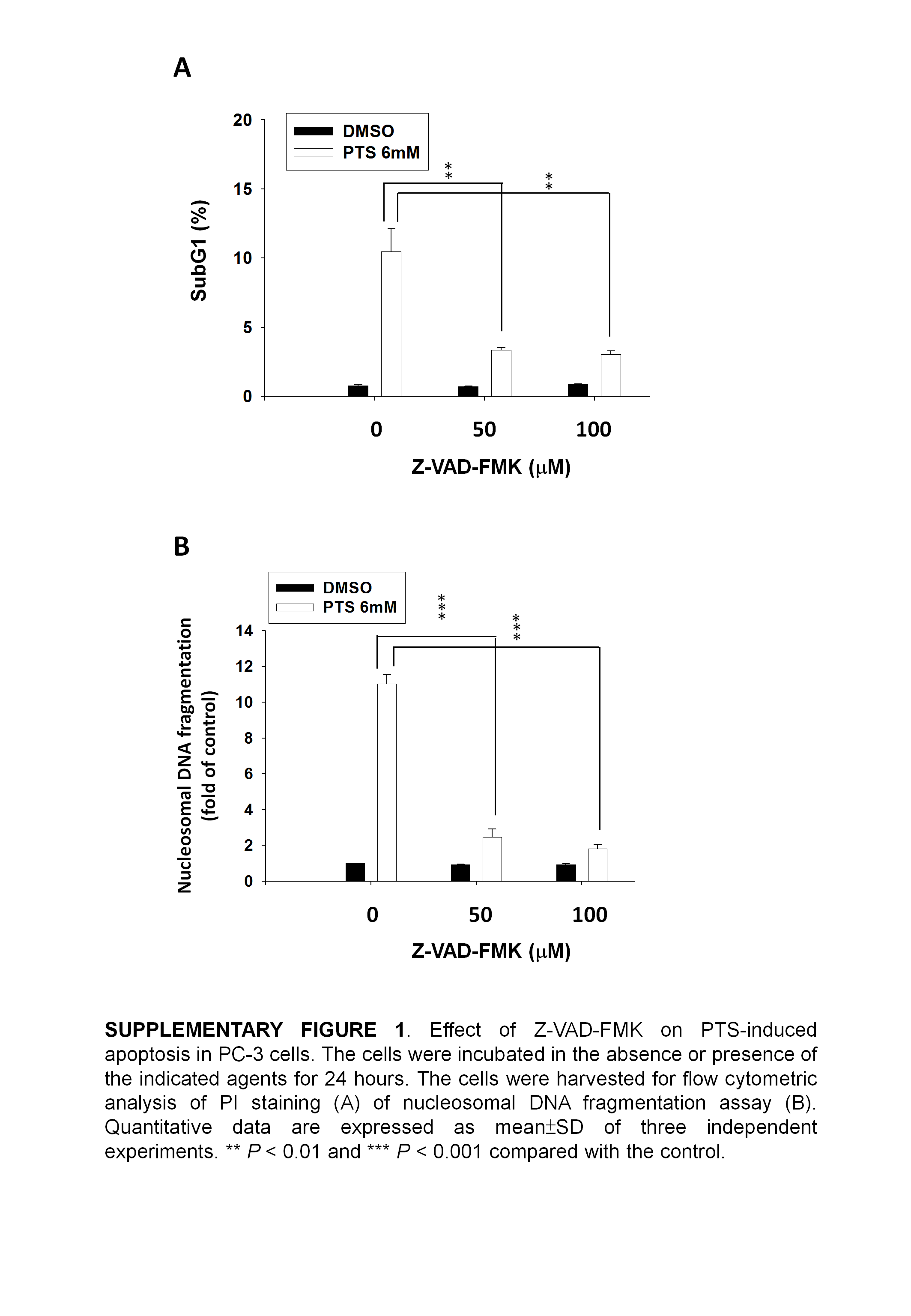

Supplement: Supplementary file 1 [file Image_1.TIF]

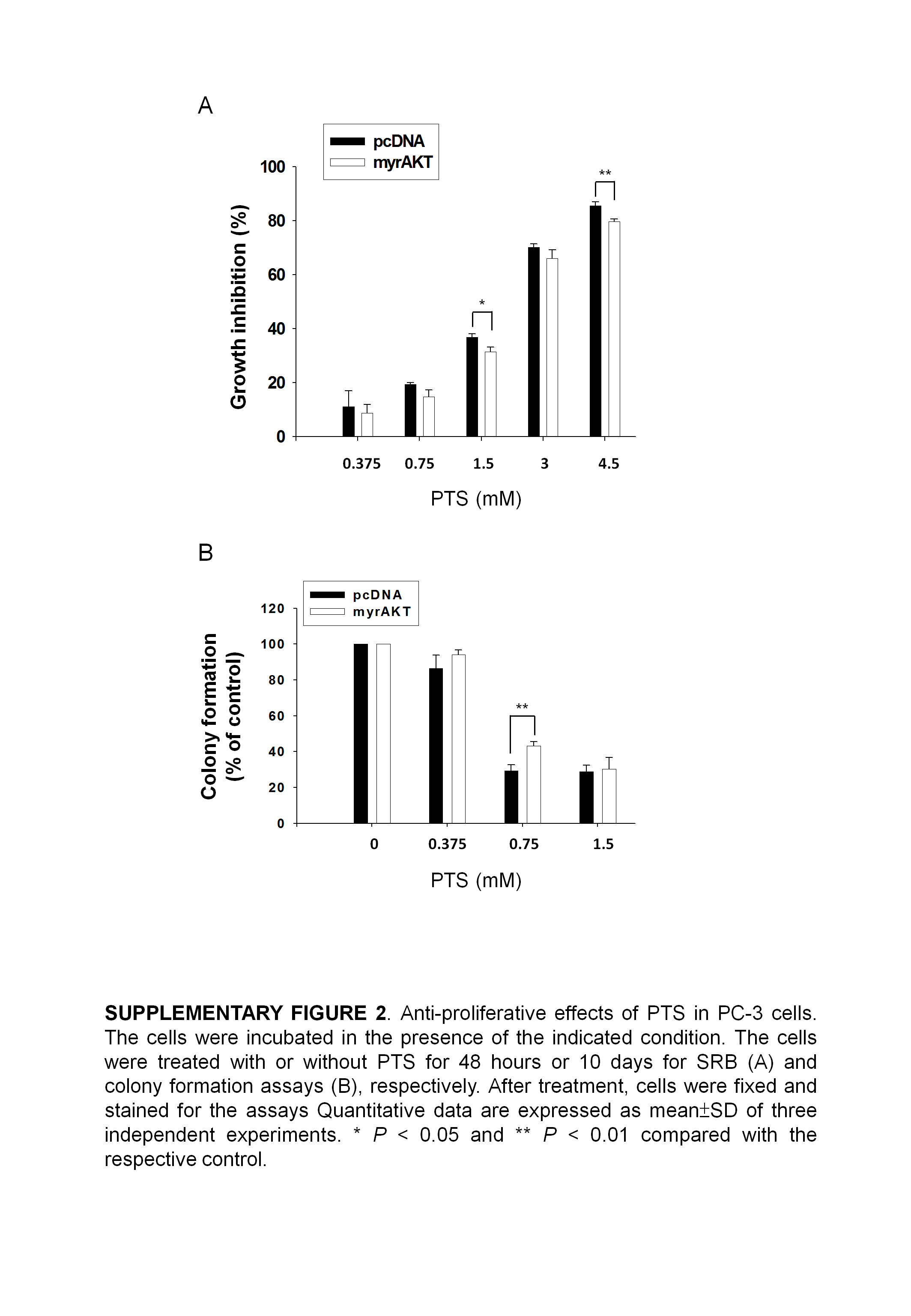

Supplement: Supplementary file 2 [file Image_2.TIF]
